# Early warning systems (EWSs) for chikungunya, dengue, malaria, yellow fever, and Zika outbreaks: What is the evidence? A scoping review

**DOI:** 10.1371/journal.pntd.0009686

**Published:** 2021-09-16

**Authors:** Laith Hussain-Alkhateeb, Tatiana Rivera Ramírez, Axel Kroeger, Ernesto Gozzer, Silvia Runge-Ranzinger

**Affiliations:** 1 Global Health, School of Public Health and Community Medicine, Institute of Medicine, Sahlgrenska Academy, University of Gothenburg, Gothenburg, Sweden; 2 Centre for Medicine and Society, Albert-Ludwigs-Universität Freiburg, Freiburg, Germany; 3 Universidad Peruana Cayetano Heredia, Lima, Peru; 4 Heidelberg Institute of Global Health, University of Heidelberg, Heidelberg, Germany; Universite de Montreal, CANADA

## Abstract

**Background:**

Early warning systems (EWSs) are of increasing importance in the context of outbreak-prone diseases such as chikungunya, dengue, malaria, yellow fever, and Zika. A scoping review has been undertaken for all 5 diseases to summarize existing evidence of EWS tools in terms of their structural and statistical designs, feasibility of integration and implementation into national surveillance programs, and the users’ perspective of their applications.

**Methods:**

Data were extracted from Cochrane Database of Systematic Reviews (CDSR), Google Scholar, Latin American and Caribbean Health Sciences Literature (LILACS), PubMed, Web of Science, and WHO Library Database (WHOLIS) databases until August 2019. Included were studies reporting on (a) experiences with existing EWS, including implemented tools; and (b) the development or implementation of EWS in a particular setting. No restrictions were applied regarding year of publication, language or geographical area.

**Findings:**

Through the first screening, 11,710 documents for dengue, 2,757 for Zika, 2,706 for chikungunya, 24,611 for malaria, and 4,963 for yellow fever were identified. After applying the selection criteria, a total of 37 studies were included in this review. Key findings were the following: (1) a large number of studies showed the quality performance of their prediction models but except for dengue outbreaks, only few presented statistical prediction validity of EWS; (2) while entomological, epidemiological, and social media alarm indicators are potentially useful for outbreak warning, almost all studies focus primarily or exclusively on meteorological indicators, which tends to limit the prediction capacity; (3) no assessment of the integration of the EWS into a routine surveillance system could be found, and only few studies addressed the users’ perspective of the tool; (4) almost all EWS tools require highly skilled users with advanced statistics; and (5) spatial prediction remains a limitation with no tool currently able to map high transmission areas at small spatial level.

**Conclusions:**

In view of the escalating infectious diseases as global threats, gaps and challenges are significantly present within the EWS applications. While some advanced EWS showed high prediction abilities, the scarcity of tool assessments in terms of integration into existing national surveillance systems as well as of the feasibility of transforming model outputs into local vector control or action plans tends to limit in most cases the support of countries in controlling disease outbreaks.

## Introduction

Epidemics of arboviral diseases transmitted by Aedes mosquitoes—such as chikungunya, dengue, yellow fever, and Zika—have emerged or reemerged over the past 5 decades, overburdening already stretched health systems. Approximately 2.5 billion people live in risk areas of Aedes-borne diseases, and, collectively, an estimated 390 million infections occur annually in about 100 countries [1–4]. Unfortunately, risk forecasts indicate that these epidemics will intensify and reach new geographical areas throughout the 21st century [5]. This fact is largely driven by a combination of urbanization, poor living conditions, international travel and trade, changes in mosquito distribution and abundance, climate variability, and climate change [6–8].

Also, malaria, an Anopheles mosquito–transmitted disease in tropical and subtropical areas, has often shown its potential for large outbreaks. This may happen in the highly endemic areas of sub-Saharan Africa but also in areas of malaria elimination in Asia and Latin America where the fading herd immunity makes people more susceptible for infections and allows local outbreaks to occur [9,10].

Vector-borne diseases can mainly be controlled through effective vector control. Even after the advent of a vaccine, as available for yellow fever, vector management will continue to be important. Only for malaria, a number of therapeutic treatment options are available [11]. Due to the high vector capacity of Aedes mosquitoes, the required level of vector control interventions to prevent transmission is usually not being achieved, and outbreaks have become increasingly frequent [11]. Data are usually provided by the routine disease surveillance systems occasionally complemented by entomological data. The information often arrives too late or is of low quality or in the wrong format [12,13]. As a result, outbreaks are usually detected too late when infections have already spread.

Forecasting disease outbreaks is highly desirable to give time to the vector control services for preparing the response. For outbreak early warning systems (EWSs), countries need standard operational procedures (SOPs) to identify consistently through alarm signals an increased outbreak risk in time and space triggering an early response. Several EWSs have been developed for our target diseases, and most of these have commonalities in their structural design, functions, and analytical approach [14–17]. However, studies addressing the effectiveness of space and time prediction and how the EWS may improve coordination among the operators at national and district level are scarce. This scoping review summarizes and discusses the evidence of different EWSs, their performance, and abilities to predict outbreaks of our target diseases, providing an overview of the state of the art and recommendations for the future development of a practical outbreak prediction tool. This review will recapitulate (1) EWS prediction validity in terms of sensitivity, specificity, positive predictive value (PPV), and negative predictive value (NPV) of outbreak warning; (2) if studies addressed the operational feasibility of the EWS in terms of user-friendliness, cost, capacity of being easily implemented within countries’ national control programs, coordination, and the requirements for increasing its efficiency; and (3) the scopes of study designs for assessing the EWS effectiveness in triggering adequate response activities.

## Methods

Methods used were predefined in a protocol based on the Preferred Reporting Items for Systematic reviews and Meta-Analyses (PRISMA) statement [18].

### Search strategy, databases, and search terms

Literature search and analysis was carried out until August 5, 2019. Data were extracted from the following databases: (1) Cochrane Infectious Diseases Group (CIDG) Specialised Register and Cochrane Central Register of Controlled Trials (CENTRAL); (2) Google Scholar; (3) Latin American and Caribbean Health Sciences Literature (LILACS); (4) PubMed; (5) Web of Science; and (6) WHO Library Database (WHOLIS). The reference lists of all included papers were screened for further relevant studies.

The inclusion criteria of articles were (1) primary research published in a peer-reviewed journal; (2) studies addressing any type of existing or developing prediction model; and (3) dealing with chikungunya, dengue, malaria, yellow fever, and Zika diseases or a combination of the diseases; presenting (a) experiences with existing EWS (stand-alone or integrated into the national surveillance systems); and (b) the development or implementation of EWS in a particular setting. EWS studies were excluded if merely investigating trends or correlations of particular alarm indicators. Studies were also excluded if they neither reported nor provided sufficient data to outline the type of mathematical model used or the temporal and spatial prediction of the tool. Further studies were excluded if they failed to demonstrate a developed or prototype of prediction model—i.e., merely presented and discussed candidate list of potential alarm indicators or studies with models constructed to elucidate transmission dynamics. No exclusion due to study design was made, except for conference abstracts, book chapters, or studies that are published by journals of a local institute.

Searches were conducted in English, assuming that most relevant studies are indexed in English, with title and abstract in English. No restrictions were applied for year of publication, geographical area, or language.

The terms “early warning,” “forecasting,” and “prediction” have been probed in a pilot search as Medical Subject Headings (MeSH) terms and as free-text terms. The terms “forecasting” and “prediction” have been discarded thereafter for the final search, as they did not show EWS in a public health sense predicting epidemics, but mostly clinical related studies predicting outcome of clinical disease.

The terms “chikungunya,” “dengue,” “malaria,” “yellow fever,” and “Zika” were used as MeSH terms and as free-text terms, depending on the function of the database. The searches were run for the combination of “early warning” AND the disease.

PubMed has been searched using MeSH terms for the diseases and free-text term for “early warning.” For Google Scholar, LILACS, and WHOLIS, free-text terms for both categories have been use. For CIDG Specialised Register and CENTRAL, the disease and “early warning” were searched as MeSH terms.

### Quality assessment and assurance

Searches were performed by the team of authors and double-checked until consistent results were found. One author (LH or EG) screened all hits for their relevance. As for Google Scholar, a large number of hits were derived (>10,000 for dengue), hits were sorted by relevance, and the first 120 hits were established as a suitable number, with no additional relevant hits encountered toward the end of the search.

Two data extractors (LH and SRR for chikungunya, dengue, yellow fever, and Zika; LH and EG for malaria) independently assessed the abstracts and full text of the preselected references for potential eligibility by applying all inclusion and all exclusion criteria.

After full agreement, data were entered into a predefined Excel data extraction form by author, title, journal, publication date, and study design as well as different outcome parameters as they emerged from the studies, following the Consolidated Standards of Reporting Trials (CONSORT) 2010 checklist [19]. Extracted parameters are listed below in detail.

Risk of bias of the included studies has not been assessed, as no systematic but a scoping review was performed, but the quality was assured by strict application of inclusion and exclusion criteria, considering several quality aspects as described above.

### Data extraction and analysis

The above described data extraction form was developed based on the study objectives and relevant predefined quantitative and qualitative outcome variables. Further categories were also considered including (1) studies addressing surveillance as structured form of information on disease cases, meteorological, epidemiological, and entomological information (indicator-based surveillance, IBS); and 2) studies on surveillance of unofficial or unstructured information (such as social media and community reports) or of social events (event-based surveillance, EBS). EWS using epidemiological, entomological, or climate data for outbreak prediction were termed “alarm-informed EWS.” EWS based on increased case numbers in comparison with historical data were termed “case-informed EWS.” Further characteristics of the studies taken into consideration were (a) methodological (case study, time series, and randomized control trials (RCT), etc.); (b) the models/tools that have been investigated (and their study scope); and (c) the disease(s) being investigated. Detailed information was extracted on specific outcomes as sensitivity, specificity, PPV/NPV outputs of the models, local coverage of the tool (central, district/province, or subdistrict level), integration (into national surveillance program), and the level of implementation (central, district, or subdistrict levels). Furthermore, the end user of the tool (e.g., public health officers, institutes, researchers, and others), the user-friendliness of the EWS tool, its temporal and spatial risk prediction and its integration feasibility including, resource needed to implement, prediction duration or timelag and, the duration of dataset were all captured in this review. The data extraction form has been further synthesized to evidence tables (Tables 1–3).

**Table 1:** ID, study design, model, and statistics used.

Article ID	Authors (year)	Types of study design	Publication year and country/region	Types of models/statistics used	Category of study*
D1 [21]	Hussain-Alkhateeb and colleagues (2018)	Retrospective; cohort	2018 Multicountry	Shewhart and endemic channel approach (moving average)	1
D2 [26]	Shi and colleagues (2016)	Retrospective analysis of surveillance data	2016 Singapore	Machine learning: absolute shrinkage and selection operator (LASSO)	1
D3 [12]	Bowman and colleagues (2016)	Retrospective analysis of surveillance data	2016 Multicountry	Shewhart and endemic channel approach (moving average)	1
D4 [31]	Ledien and colleagues (2019)	Retrospective, Cross-sectional study design	2019 Cambodia	Bayesian algorithms to detect outbreaks using count data series	2
D5 [28]	Zhang and colleagues (2014)	Prospective analysis of a real-world system	2014 China	Time series moving percentile method based on historical data	2
D6 [27]	Chen and colleagues (2018)	Retrospective analysis of surveillance design	2018 Singapore	Derive dynamic risk maps for dengue transmission. LASSO-based regression	1
D7 [57]	Ramadona and colleagues (2016)	Retrospective analysis of surveillance data	2016 Indonesia	Generalized linear regression models with a Gaussian link of disease	2
D8 [30]	Ortiz and colleagues (2015)	Retroprospective time series analyses	2015 Cuba	Linear models, probability distributions or time series	2
D9 [38]	Lee and colleagues (2017)	Retrospective analysis of surveillance data	2017 Colombia	Nonlinear regression model	2
D10 [23]	Semenza (2015)	Retrospective analysis of surveillance data	2015 Europe	Hierarchical multivariate model	2
D11 [43]	Sang and colleagues (2015)	Retrospective analysis of surveillance data	2014 China	STL	2
D12 [41]	Zhang and colleagues (2016)	Retrospective analysis of surveillance data	2016 China	A negative binomial regression model with a log link function	2
D13 [42]	Li and colleagues (2017)	Retrospective analysis of surveillance data	2017 China	GAMs	2
D14 [44]	Adde and colleagues (2016)	Retrospective analysis of surveillance data	2016 French Guiana	Lagged correlations and composite analyses	2
D15 [40]	Eastin and colleagues (2014)	Retrospective analysis of surveillance data	2014 Colombia	ARIMA model	2
D16 [45]	Guo and colleagues (2017)	Retrospective analysis of surveillance data	2017 China	Several machines learning algorithms (LASSO) linear regression model and GAM	2
D17 [13]	Hii and colleagues (2012)	Retrospective analysis of surveillance data	2012 Singapore	Poisson multivariate regression model and autoregression	2
D18 [35]	Wongkoon and colleagues (2012)	Retrospective analysis of surveillance data	2012 Thailand	SARIMA	2
D19 [32]	Putra and colleagues (2017)	Mathematical simulation modeling	2017 Indonesia	Model was developed using logistic regression	2
D20 [33]	Hidayati and colleagues (2012)	Mathematical simulation modeling	2012 Indonesia	Stochastic spreadsheet model	2
D21 [39]	Halide and colleagues (2008)	Retrospective analysis of surveillance data	2008 Indonesia	Linear multiple regression model	2
D22 [58]	Lowe and colleagues (2011)	Retrospective analysis of surveillance data	2011 Brazil	A negative binomial model formulation extra-Poisson variation (Bayesian framework) using MCMC	2
D23 [37]	Yu and colleagues (2011)	Retrospective analysis of surveillance	2011 Taiwan	Stochastic BME	2
D24 [29]	Lowe and colleagues (2016)	Prospective surveillance design	2016 Brazil	Bayesian spatiotemporal model	2
D25 [69]	Lowe and colleagues (2014)	Retrospective analysis of surveillance data	2014 Brazil	Spatiotemporal hierarchical Bayesian model	2
D26 [36]	Lowe and colleagues (2013)	Retrospective analysis of surveillance data	2013 Brazil	Spatiotemporal generalized linear mixed model with parameters estimated in a Bayesian framework	2
D27 [56]	Withanage and colleagues (2018)	Retrospective analysis of surveillance data	2018 Sri Lanka	Time series regression model	2
D28 [22]	Chen and colleagues (2018)	Retrospective analysis of surveillance data	2018 Multicountry	Machine learning LASSO method	2
Z1 [24]	Teng and colleagues (2017)	Retrospective analysis of surveillance data	2017 PAHO	ARIMA model	2
Z2 [25]	Chien and colleagues (2018)	Retrospective analysis of surveillance data	2018 Colombia	Generalized linear model with additional cross-basis functions	2
M1 [47]	Githeko and colleagues (2001)	Prospective surveillance design	2001 Kenya	Vector capacity and malaria epidemic prediction model (additive model)	2
M2 [48]	Githeko and colleagues (2014)	Retrospective analysis of surveillance data	2014 Multicountry	Additive, multiplicative, and +18°C models	2
M3 [34]	Githeko and colleagues (2018)	Retrospective analysis of surveillance data	2018 Kenya	Additive and multiplicative models	1
M4 [49]	Midekisa and colleagues (2012)	Prospective surveillance design	2012 Ethiopia	ARIMA models (SARIMA)	2
M5 [50]	Smith and colleagues (2017)	Prospective surveillance design	2017 Solomon Islands	SCOPIC	2
M6 [51]	Ruiz and colleagues (2006)	Prospective surveillance design	2006 Colombia	A combination of parasite transmission, simulation of vector ecology, behavior patterns, and dynamics of mosquitoes	2
M7 [46]	Merkord and colleagues (2017)	Retrospective validation surveillance data	2017 Ethiopia	Time series models	2

*1 = experience with existing EWS and 2 = EWS exercise.

Reference: Article ID; D = dengue, Z = Zika, and M = malaria.

ARIMA, autoregressive integrated moving average; BME, Bayesian maximum entropy; EWS, early warning system; GAM, generalized additive model; MCMC, Markov chain Monte Carlo; PAHO, Pan American Health Organization; SARIMA, seasonal autoregressive integrated moving average; SCOPIC, seasonal climate outlooks in Pacific Island countries; STL, seasonal-trend decomposition procedure based on loess.

**Table 2:** Characteristics of EWARS systems under investigation.

Article ID	Case- or alarm-informed EWS	IBS or EBS	Resource needed to implement and use	Outbreak indicator	Alarm indicators	Source of data
D1 [21]	Alarm informed	IBS	Routine access to data, staff training (suitable for unskilled)	Weekly hospitalized cases	Multiple of meteorological, epidemiological, and entomological variables	National surveillance system and local meteorological stations
D2 [26]	Alarm informed	IBS	Routine access to data, staff training (suitable for unskilled)	Weekly reported cases	Multiple of meteorological, epidemiological, and entomological variables	National surveillance system, local meteorological stations, and Department of Statistics for the demographics
D3 [12]	Alarm informed	IBS	Routine access to data, staff training (suitable for unskilled)	Weekly probable and hospitalized cases	Multiple of meteorological, epidemiological, and entomological variables	National surveillance system and local meteorological stations
D4 [31]	Case informed	IBS	Routine access to data, staff training (suitable for unskilled)	Weekly probable and lab-confirmed cases	Predictive distribution of provincial weekly reported cases	National surveillance system
D5 [28]	Case informed	IBS	Routine access to data, staff training (suitable for unskilled)	Biweekly suspected, and lab-confirmed cases	Predictive distribution of provincial weekly reported cases	CDC
D6 [27]	Alarm informed	IBS	Routine access to data (users’ training and level of skills not discussed)	Weekly confirmed or lab-confirmed cases	Weekly meteorological information	MOH and the Centre for Remote Imaging, Sensing, and Processing
D7 [57]	Alarm informed	IBS	Routine access to data (users’ training and level of skills not discussed)	Lab-confirmed cases	Meteorological and number of cases	Provincial surveillance system and local meteorological stations
D8 [30]	Alarm informed	IBS	Routine access to data (users’ training and level of skills not discussed)	Mathematically simulated: infestation index of *Aedes aegypti*	Extensive meteorological data	National surveillance system and local meteorological stations
D9 [38]	Alarm informed	IBS	Routine access to data (users’ training and level of skills not discussed)	Monthly incidence cases	Multiple of meteorological, epidemiological, and remote sensing data	National surveillance system
D10 [23]	Alarm informed	IBS	Not discussed	Imported cases	Monthly meteorological information	The European environment and epidemiology (E3) network
D11 [43]	Alarm informed	IBS	Routine access to data, staff training (users’ skills not discussed)	Lab-confirmed cases	Monthly meteorological information	National surveillance system
D12 41]	Alarm informed	IBS	Not discussed	Weekly notifiable cases	Meteorological information	National CDC, provincial, meteorological and demographic data
D13 [42]	Alarm informed	IBS	Routine access to data and high statistical skills	Weekly notifiable cases	Meteorological information	National surveillance, meteorological systems, and search engine of Baidu index database
D14 [44]	Alarm informed	IBS	Routine access to time series data and high statistical skills	Weekly confirmed cases	Meteorological information	Local Arbovirus National Reference Centre
D15 [40]	Alarm informed	IBS	Routine access to time series data and high statistical skills	Lab-confirmed cases	Meteorological information	National surveillance system and the Global Historical Climate Network
D16 [45]	Alarm informed	IBS	Routine access to time series data and high statistical skills	Lab-confirmed cases	Weekly meteorological information	National surveillance and meteorological systems
D17 [13]	Alarm informed	IBS	Not discussed	Lab-confirmed cases	Weekly meteorological information	MOH, national climatic, and National Oceanic and Atmospheric Administration
D18 [35]	Case informed	IBS	Routine access to time series data and high statistical skills	Monthly confirmed cases	Historical cases	National surveillance system and MOPH
D19 [32]	Case informed	IBS	Routine access to time series data and high statistical skills	Monthly lab-confirmed cases	Index of mosquito survival and disease resistance (entomological data)	Health department and the Center for Climate data
D20 [33]	Alarm informed	IBS	Routine access to time series data and high statistical skills	Dengue cases	Information of dengue incidence and meteorological information	MOH
D21 [39]	Alarm informed	IBS	Routine access to time series data and high statistical skills	Monthly confirmed cases	Historical cases and meteorological information	MOH and WMO
D22 [58]	Alarm informed	IBS	Routine access to time series data and high statistical skills	Lab-confirmed cases	Meteorological information	National surveillance system and cartographic data
D23 [37]	Alarm informed	IBS	Routine access to time series data and high statistical skills	Lab-confirmed cases	Meteorological and entomological information	National surveillance system and local entomological and meteorological stations
D24 [29]	Alarm informed	IBS	Routine access to time series data and high statistical skills	Dengue cases	Meteorological and entomological information	Surveillance system (MOH)
D25 [69]	Alarm informed	IBS	Routine access to time series data and high statistical skills	Dengue cases	Meteorological information	Surveillance system (MOH) and ECMWF
D26 [36]	Alarm informed	IBS	Routine access to time series data and high statistical skills	Monthly confirmed cases	Meteorological, entomological, cartographic, and epidemiological information	National surveillance, meteorological systems, and Institute for Geography and Statistics
D27 [56]	Alarm informed	IBS	Routine access to time series data and high statistical skills	Monthly confirmed cases	Meteorological information	Regional epidemiology and meteorological stations
D28 [22]	Alarm informed	IBS	Routine access to time series data and high statistical skills	Monthly confirmed cases	Meteorological information	Local MOH and meteorological stations (Japan, Singapore, Taiwan, and Thailand)
Z1 [24]	Alarm informed	EBS	Routine access to time series data and Google Trends search data and high statistical skills	Confirmed and suspected cases	Google Trends search	Google, national surveillance data, and PAHO
Z2 [25]	Alarm informed	IBS	Routine access to time series data and high statistical skills	Suspected cases	Meteorological information	National surveillance system and local meteorological stations
M1 [47]	Alarm informed	IBS	Routine access to time series data and high statistical skills	Hospitalized and lab-confirmed cases	Meteorological information	International Research Institute for Climate Prediction
M2 [48]	Alarm informed	IBS	Routine access to time series data	Lab-confirmed cases	Meteorological information	Meteorological stations
M3 [34]	Alarm informed	IBS	Routine access to time series data	Lab-confirmed cases	Meteorological information	MOH
M4 [49]	Alarm informed	IBS	Routine access to time series data and high statistical skills	Lab-confirmed cases	Meteorological information, vegetation indices, and actual evapotranspiration	Satellite-derived meteorological data and earth sciences data
M5 [50]	Alarm informed	IBS	Routine access to time series data and high statistical skills	Monthly confirmed cases	Meteorological information	N/A
M6 [51]	Alarm informed	IBS	Routine access to time series data and high statistical skills	Model simulation (no cases)	Meteorological information	Local meteorological and national surveillance
M7 [46]	Case informed	EBS	Routine access to time series data and high statistical skills	Incidence of malaria	N/A	ARHB US NASA

Reference: Article ID; D = dengue, Z = Zika, and M = malaria.

ARHB, Amhara Regional Health Bureau; CDC, Centre for Disease Control and Prevention; EBS, event-based surveillance; ECMWF, European Centre for Medium-Range Weather Forecast; EWARS, early warning and response system; IBS, indicator-based surveillance; lab, laboratory; MOH, Ministry of Health; MOPH, Ministry of Public Health; N/A, not available/no answer; NASA, National Aeronautics and Space Administration; PAHO, Pan American Health Organization; WMO, World Meteorological Organization.

**Table 3:** Main findings, conclusions, and limitations of the studies.

Article ID	Main study findings, including prediction quality (sensitivity + PPV)	Temporal and spatial risk prediction	Prediction time lag	Study or model limitations	Conclusions
D1 [21]	Improved prediction, user-friendly, implementable tool. Sensitivity: 50%–100% and PPV: 40%–88%	High temporal prediction, low spatial prediction (district level)	A range of 1–12 weeks	Lacks predictions at small spatial unit and requires weekly to enable operational forecasting	The tool is pragmatic and useful for detecting imminent outbreaks
D2 [26]	Operationally useful, LASSO was superior to methods (SARIMA model) except the first 2-week window	High temporal precision, low spatial prediction (district level)	12 weeks	LASSO methods are not amenable to interpretation (mainly at longer forecasting window), hindered by the numerous covariates acting at different lags	Automated machine learning methods such as LASSO can markedly improve forecasting techniques
D3 [12]	Sensitivities: 72%–97% and PPV: 45%–86% at a lag of 1–12 weeks	High temporal precision, low spatial prediction (district level)	1–12 weeks	Can be disturbed by inconsistent and missing data especially with regard to entomological indices	Probable cases and meteorological variables indicate for increased risk of transmission
D4 [31]	Sensitivity: 50%–100% and specificity: 75%–100%	High temporal precision, low spatial prediction (district level)	5 weeks	Algorithm used needs to be trained, which may cause a loss of robustness if the outbreak pattern changes or differs significantly from previous years	Surveillance R-package algorithms are free and implementable. Time-space trends monitoring can also be useful
D5 [28]	Sensitivity: 100% and specificity: 99.8% and a median time to detection of 3 days	Low temporal prediction but high spatial prediction	3 days	N/A	CIDARS had good sensitivity, specificity and timeliness of outbreak detection
D6 [27]	AUCs are 75% for forecasting 12 weeks and 80% for 5 weeks in advance	High temporal and high spatial predictions	1–12 weeks	The model is highly reliant on a rich dataset of georeferenced case identifications and demand regular update and the adaptation require pre-adjustments to the grid used in different geo-areas.	Spatially resolved forecasts of geographically structured diseases can be obtained at a neighborhood level in urban and rural environments for guiding control efforts
D7 [57]	A combination of surveillance and meteorological was optimal; temperature at lag 3 weeks, rainfall at lag 2 weeks, and rainfall at lag 3 weeks. Sensitivity: 88.9%, specificity: 81.0%, PPV: 74.4%, and NPV: 92.2%	High temporal level but low spatial prediction	12 weeks	Predictive model could explain only 64% of the variation in the occurrence of cases and is biased by underreporting of cases	Past disease incidence data, up to years, are crucial predictive possibly indicating cross-immunity status of the population
D8 [30]	Models for describing, simulating, and predicting spatial patterns of *Aedes aegypti* populations associated with climate variability patterns	Unknown temporal but low spatial prediction	N/A	N/A	Using indices of climate variability can construct spatial models providing warning of potential changes in vector populations in rainy and dry seasons and by months
D9 [38]	Sensitivity: 75% (lag, 1–5 months) and PPV: 12.5%. Climate predictors were good classifiers of risk areas based on the different climate in different regions	High temporal and high spatial predictions	4–52 weeks	The model is limited to issuing alerts with short-time intervals (1–5 months ahead), which may not be practical in operational modes	It is possible to detect dengue outbreaks ahead of time and identify populations at high risk
D10 [23]	A 9% increase in the incidence of imported cases for every additional 10,000 travelers arriving from affected areas	No temporal prediction but low spatial prediction	N/A	N/A	The risk of disease importation was computed with the volume of international traveler from disease-affected areas worldwide
D11 [43]	Time series Poisson model using climate data well predicted at time lag of 3 months after controlling the autocorrelation, seasonality, and long-term trend	High temporal but low spatial prediction	48–96 weeks	N/A	Transmission vector *Aedes albopictus*, imported cases, monthly climatic information are useful for cheap and effective EWS
D12 [41]	Sensitivity/specificity: 78%/92% for a threshold of 3 cases per week, sensitivity/specificity: 91%/91% for a threshold of 2 cases per week, and sensitivity/specificity: 85%/87% for a threshold of 1 case per week	Low temporal prediction but no spatial prediction	1 week	Limited to climatic factors and can be biased by underreporting of cases	Occurrence of outbreaks in the study city could impact disease outbreaks in neighboring city under suitable weather conditions
D13 [42]	Models with DBSI (ICC: 0.94 and RMSE: 59.86) is better than the model without (ICC: 0.72 and RMSE: 203.29)	Low temporal but poor or no spatial prediction	1 week	Uses short-term time series data and prone to confounding effect	DBSI combined with traditional disease surveillance and meteorological data can improve the dengue EWS
D14 [44]	Summer Equatorial Pacific Ocean sea surface temperatures and Azores high sea-level pressure model correctly predicted 80% and missed 15% of the nonepidemic years	Low temporal and Low spatial prediction	Annual (year-to-year variability)	N/A	Outbreak resurgence can be modeled using a simple combination of climate indicators
D15 [40]	Environment-based, multivariate, autoregressive models predicted 2–26 weeks ahead	High temporal and Low or no spatial prediction	8–26 weeks	N/A	Outbreaks often occurred when extreme daily temperatures are confined within the 18–32°C range, Patterns of spatial variability across endemic regions may be related to variations in the built environment, ecology, local weather, population density, mitigation efforts, and host mobility
D16 [45]	SVR model selected by a cross-validation technique accurately forecasted at 12 weeks with smallest prediction error	High temporal but low or no spatial prediction	12 weeks	Internet searching behavior is susceptible to the impact of media reports, which may affect the performance of the model	SVR model achieved a superior performance in comparison with other forecasting techniques
D17 [13]	The model predicted accurately with <3% false alarm	Good temporal but poor or no spatial prediction	16 weeks	N/A	Models using temperature and rainfall could be simple, precise, and low-cost tools for disease forecasting
D18 [35]	SARIMA model is robust and autoregression, moving average and seasonal moving average are key determinants of transmission	Low temporal and low spatial prediction	Annual	Long history of data is required and a sophisticated analysis that requires a skilled user	SARIMA has great potential to be used as a decision supportive tool due to its ability to predict when and where
D19 [32]	Ratio of basic offspring number and basic reproductive ratio is considered outbreak if > = 0.5	Low temporal and low spatial predictions	N/A	Warrant for more assessment for increasing its sensitivity	Model simulations show that mosquito population are more affected by weather factors than human
D20 [33]	Climate factor and incidence rate of dengue before prediction period were superior to rainfall index of week-n	High temporal and low spatial prediction	2–7 weeks	N/A	The provision of both structure and infrastructure is recommended to be in line with incidence rate prediction value
D21 [39]	The model has useful only up to lead 6 times, i.e., correlation >0.5, and as the lead times increase, the match between prediction and observation deteriorates	High temporal but low spatial prediction	4–24 weeks	Requires long historical data for the evaluation	The model is well suited due to its simplicity in data requirement and computational effort
D22 [58]	Predictions are improved both spatially and temporally when using the GLMM; sensitivity of 83% and false alarm of 8%	high temporal and low spatial prediction	12 weeks	Fails to capture the temporal variability in case counts (due to population immunity to the dominant circulating serotype or specific health interventions)	Seasonal climate forecasts could predict incidence months in advance
D23 [37]	Models link outbreaks and climatic conditions and yielded 1-week lag based on spatiotemporal predictions	High temporal and low spatial prediction	8–12 weeks	N/A	SBME is valuable to timely identify, control, and efficiently prevent disease spreading in time and space
D24 [29]	The model was superior (sensitivity: 57%) to the null model (33%)	High temporal and low spatial prediction	4–12 weeks	N/A	Incorporating real-time seasonal climate forecasts and epidemiological data is beneficial for prediction
D25 [69]	The rank probability skill score RPSS was superior to the benchmark with AUC of 0.86 for temperature and 0.84 for rainfall	High temporal and low spatial prediction	12 weeks	N/A	Close collaboration between public health specialists, climate scientists, and mathematical modelers is crucial for successful implementation of seasonal climate forecasts
D26 [36]	The prediction improved with applying criterion >50% chance of exceeding 300 cases per 100,000 inhabitants, with false alarm: 25%	High temporal and low spatial prediction	12 weeks	N/A	Visualization technique to map ternary probabilistic forecasts can identify areas where the model predicts with certainty a particular disease risk category
D27 [56]	The model detected 5 and rejected 14 within 24 months. The Pierce skill score was 0.49, with AUC: 86% and sensitivity: 92%	Low temporal and low spatial prediction	Annual (year-to-year variability)	There is no proper mechanism to track commute-related infections to neighboring districts	Depending upon climatic factors, the previous month’s disease cases had a significant effect on disease incidences of the current month
D28 [22]	Sensitivity: 75% at 5 weeks but less sensitive to the outbreak size. Prediction improves when climatic variables and incidence in regions further away from the equator.	High temporal and low spatial prediction	1–5 weeks	Prediction accuracy might improve if incidence and weather information can be collected at a finer resolution	Short-term LASSO models predictions perform better than longer-term predictions, encouraging public health agencies to respond at short-notice to early warnings
Z1 [24]	Integer-valued autoregression is useful predictive model and enhanced by incorporating Google Trends data	High temporal and low spatial prediction	1–12 weeks	N/A	Accessible and flexible dynamic forecast model can advance early warning prediction
Z2 [25]	Average humidity, total rainfall, and maximum temperature were best meteorological factors with prediction lag between 15 and 20 weeks	High temporal and low spatial prediction	4–20 weeks	The interaction term between the nonlinear smoothing function of time and the spatial function is unavailable in the model, so a real spatiotemporal pattern was unable to be investigated in this study	Meteorological factors are useful for predicting ZIKV epidemics
M1 [47]	The model was able to predict both El Niño and non-El Niño malaria outbreaks with high specificity and sensitivity	High temporal prediction	3–4 months	Data may not be readily available at the district level, and it may not be site specific	Rainfall and unusually high maximum temperatures and the number of inpatient malaria cases 3–4 months later provide a good prediction model
M2 [48]	Additive model are most suited for poorly drained U-shaped valley ecosystems while the multiplicative model was most suited for the well-drained V-shaped valley ecosystem	High temporal prediction	2–4 months	N/A	Additive and multiplicative models are designed for use in the common, well-, and poorly drained valley ecosystems
M3 [34]	The models indicated that climate variability remains a major driver of malaria epidemics	High temporal prediction	2–4 months	N/A	The multiplicative model maintained consistent prediction due to stakeholders’ confidence
M4 [49]	Malaria cases exhibited positive associations with LST at a lag of 1 month and positive associations with indicators of moisture at lags between 1 and 3 months	High temporal and low spatial prediction	1–3 months	Requires weekly rather than monthly intervals, to enable operational forecasting	Integrating modeling approaches based on historical case data (early detection) and environmental data (early warning) can enhance the effectiveness of risk forecasting
M5 [50]	Only rainfall had a consistently significant relationship with malaria	High temporal and low spatial prediction	1–6 months	N/A	Rainfall provides the best predictor of malaria transmission
M6 [51]	Temperature is the most relevant climatic parameter thus. Sporogonic and gonotrophic cycles showed to be key entomological variables controlling the transmission	High temporal prediction	1 month	Too many variables and phases that make it difficult to set in place on daily basis	Environmental factors and climate variability can be merged with selected mathematical tools (statistical and biological/eco-epidemiological models) for improved prediction tool
M7 [46]	Within early detection window (the past 6 weeks) and an early warning forecast window (the upcoming 4 weeks), the mean observed or forecasted incidence was classified as being above the mean outbreak threshold, between the mean threshold and the mean expected incidence, or below the mean expected incidence	High temporal and spatial prediction	1–4 weeks	N/A	Malaria surveillance data and environmental monitoring data can be integrated to enable near real time malaria forecast in the Amhara region

Reference: Article ID; D = dengue, Z = Zika, and M = malaria.

AUC, area under the curve; CIDARS, China Infectious Disease Automated-alert and Response System; DBSI, Dengue Baidu Search Index; EWS, early warning system; GLMM, generalized linear mixed model; ICC, intraclass correlation coefficient; LASSO, least absolute shrinkage and selection operator; LST, land surface temperature; N/A, not available/no answer; NPV, negative predictive value; PPV, positive predictive value; RMSE, root mean squared error; RPSS, rank probability skill score; SARIMA, seasonal autoregressive integrated moving average; SBME, stochastic Bayesian maximum entropy; SVR, support vector regression; ZIKV, Zika virus.

### Evaluation criteria

Prediction models are typically evaluated according to their statistical and operational performance. Statistically, the sensitivity, specificity, PPV, or NPV measures are used to quantify the quality of prediction performance of the model. Based on common recommendations of appropriate cutoff for model performance, we attributed a statistical measure of >50% to high performance prediction models [20]. The spatial prediction is crucial in the context of vector control and action plan; spatial prediction alone is less useful and must be combined with the temporal model for effective public health response. Accordingly, we defined high temporal prediction by a prediction window of 1 to 12 weeks (allowing sufficient time between prediction and action taken). We defined high spatial prediction by the prediction of hotspots within geographically defined areas of relatively small spatial scale such as villages, neighborhood, or household levels in contrast to a resolution within district scale.

## Results

### Study characteristics

**Fig 1:**
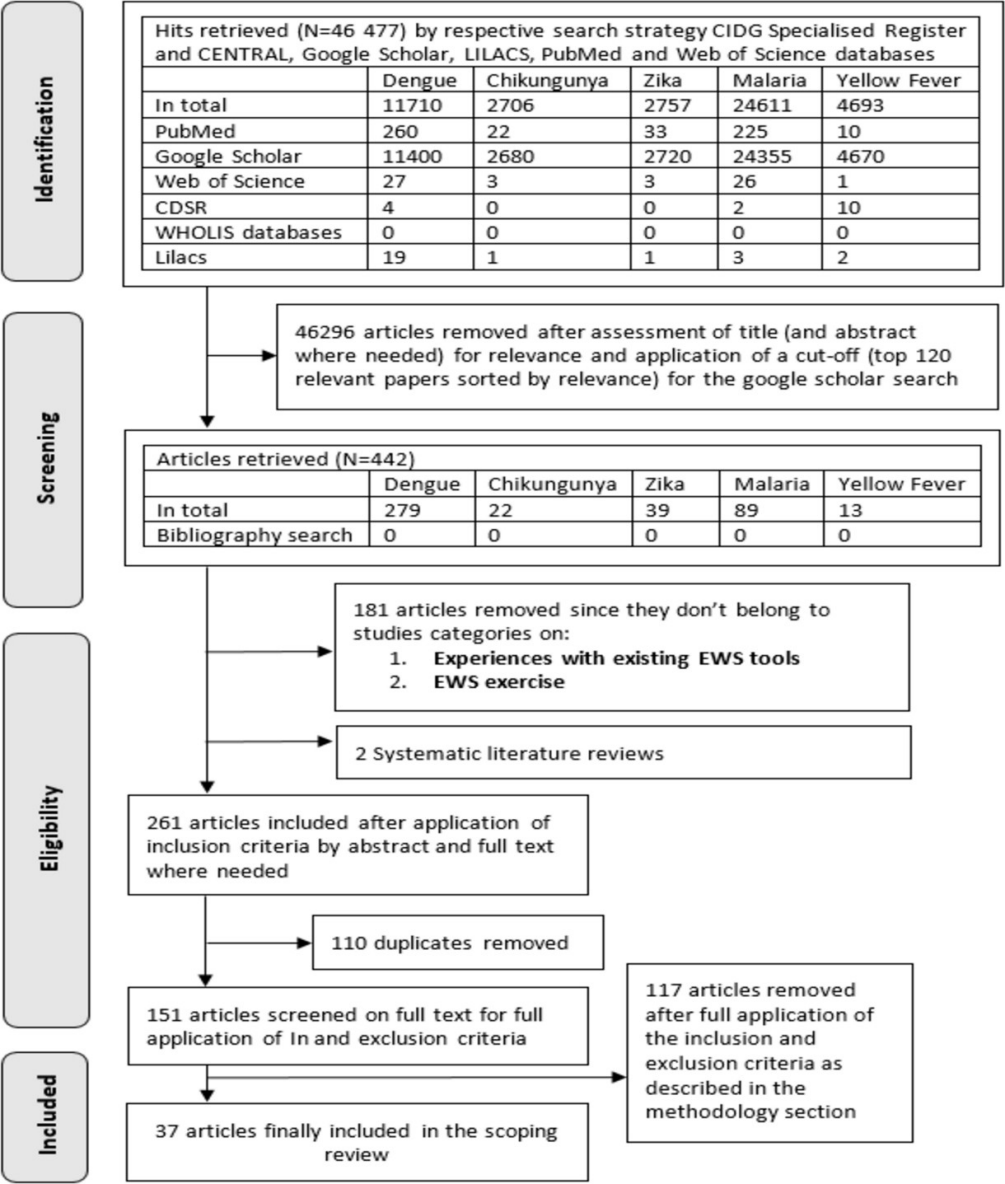
Screening and selection of articles for a scoping review on EWS. CDSR, Cochrane Database of Systematic Reviews; CENTRAL, Cochrane Central Register of Controlled Trials; CIDG, Cochrane Infectious Diseases Group; EWS, early warning system; LILACS, Latin American and Caribbean Health Sciences Literature.

A total of 46,477 hits were obtained, with 11,710 studies focused on dengue, 2,757 on Zika, 2,706 on chikungunya, 24,611 on malaria, and 4,693 on yellow fever. After review by title and abstract as well as the application of a cutoff after 120 Google Scholar hits, sorted by relevance, 279 dengue, 22 chikungunya, 39 Zika, 89 malaria, and 13 yellow fever articles were further assessed. Based on the inclusion criteria and removing of duplicates, 151 articles were assessed as full texts, but exclusion criteria finally retained 37 articles including 28 for dengue, 2 for Zika, and 7 for malaria. Fig 1 summarizes the search process.

All of the 37 studies were published between 2001 and 2019, of which 33 between 2011 and 2018. Of the 28 dengue studies, 3 [12,21,22] were conducted across different countries (multicounty) and 16 in Asia (5 in China, 4 in Indonesia, 3 in Singapore, and 1 each in Cambodia, Sri Lanka, Taiwan, and Thailand). Another 8 studies were conducted in the Americas (4 in Brazil, 2 in Colombia, 1 in Cuba, and 1 in French Guiana) and 1 [23] in Europe. The 2 Zika studies were performed in the Americas [24,25].

**Fig 2:**
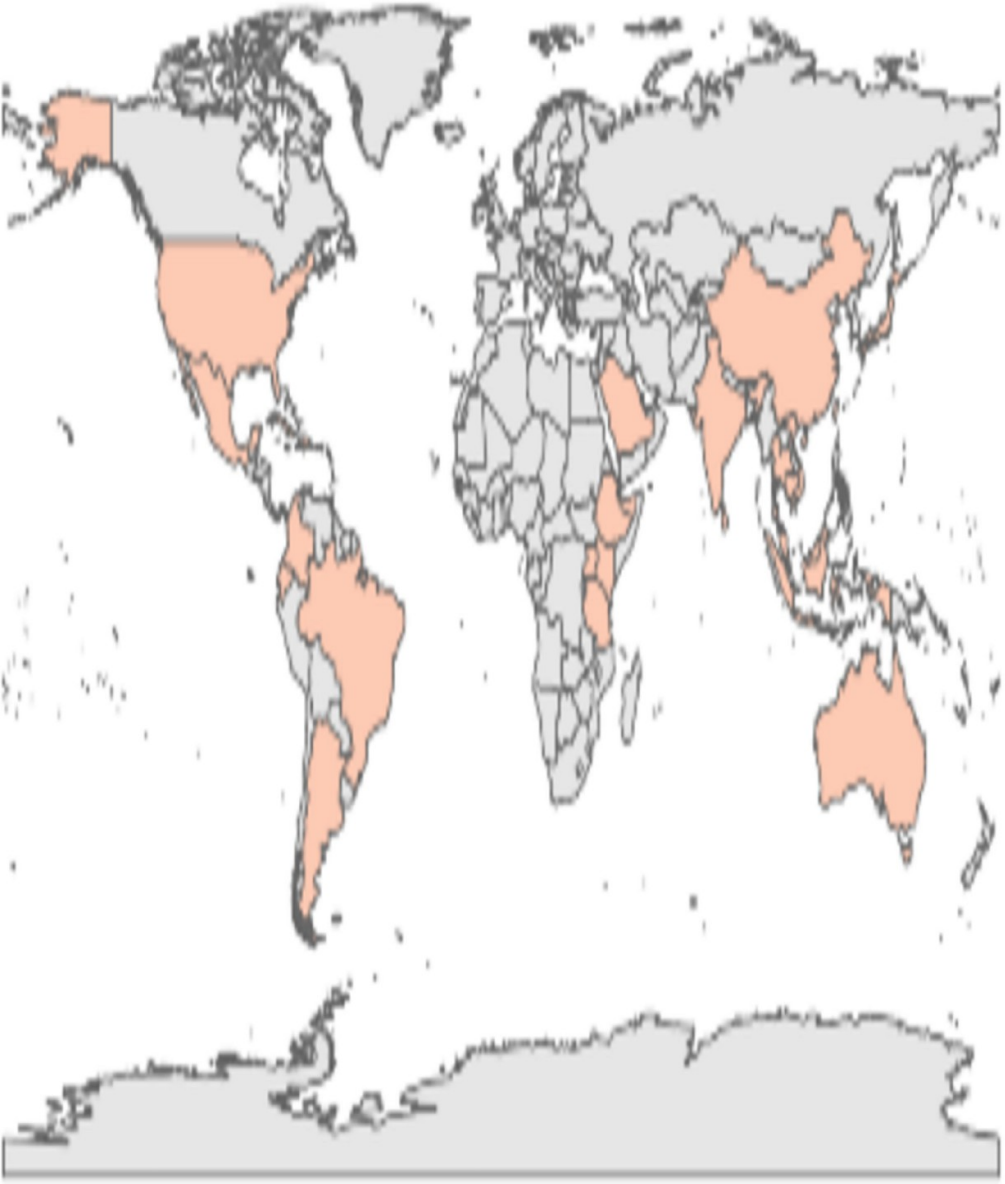
Geographic distribution of included studies.

From the 7 malaria studies that were retrieved, 1 was conducted in Colombia and 6 in Africa (2 in Ethiopia and Kenya, 1 in Tanzania, and 1 in Uganda) and 1 in the Solomon Islands in the Pacific. For details of the papers, see Fig 2 and S1 Text (country distribution of included studies).

Of the 28 studies addressing EWS for dengue outbreaks, only 4 studies presented the user perspective on implementing EWS within national programs [12,21,26,27], and 24 studies showed EWS under development (i.e., model exercise); see Table 1. Only 2 dengue studies used a prospective study design [28,29], and one applied both prospective and retrospective approaches [30]; the other 23 were retrospective analyses of surveillance data (1 of those specified as cohort study [21], 1 with a cross-sectional design [31], and 1 as a time series analysis [30]), and 2 studies were exclusively relying on mathematical simulation models [32,33]. The 2 Zika studies reported on exercises of testing EWS [24,25]. The 7 EWS malaria studies included only one existing EWS [34], while 6 other studies reported on potential EWSs or under development. The studies used a large variety of statistical methods as summarized in Table 1.

### EWS features per disease

Outbreak indicators of EWSs varied considerably across the selected studies, and, hence, we stratified the findings of the relevant outcomes by the 3 diseases (no studies were retrieved for chikungunya and yellow fewer) that were ultimately retained from the search. In line with the respective case definition used in the diverse reporting systems across national surveillance programs and between diseases, the outbreak indicators were grouped as hospitalized, laboratory-confirmed (including “reported,” “confirmed,” or “imported from neighboring districts”), and suspected cases (including “probable” cases as they were often not distinguished from “suspected”) as well as case numbers resulting from mathematical modeling (no real cases but estimates based on other covariates). The alarm indicators were categorized as entomological (including an index of mosquito survival), meteorological (including the El Niño Southern Oscillation [ENSO] index and others), epidemiological (including sociodemographic information), and social media (such as “Google Trends”).

## Study results

### Dengue

#### EWS characteristics and features

All 28 dengue studies relied on IBS, of which 24 studies were alarm informed, i.e., of outbreak predictive nature—using at least 1 meteorological, entomological or epidemiological indicator—and 4 studies [28,31,32,35] were case-informed EWS, i.e., of early outbreak detection—relying solely on previous trends of cases for outbreaks. Almost all studies demanded routine access to data as well as advanced statistical and analytical skills, but 5 studies [12,21,26,28,31] reported prediction tools that are adapted for less skilled users. The outbreak indicators used were hospitalized cases (2 studies) [12,21], laboratory-confirmed cases (23 studies), or suspected cases (3 studies) [12,28,31]. One study [30] used mathematically simulated cases. All cases were reported by the surveillance system on either weekly or monthly basis, and, occasionally, data were obtained directly from the Ministry of Health (MOH).

The dengue studies applied a spectrum of alarm indicators using meteorological (24 studies), entomological (7 studies) [12,21,26,29,32,36,37], epidemiological (5 studies) [12,21,26,36,38], and one study [36] used a cartographic indicator—these are nonclimatic indicators of altitude and aggregated census data related to urban setting.

With reference to the alarm indicators, 13 studies reported the use of data from local/regional meteorological stations, whereas 3 studies referred to international sources such as the “World Meteorological Organization” [39], the “Global Historical Climate Network” [40], and the “National Oceanic and Atmospheric Administration” [13]. Furthermore, one study [23] used data from the regional Environment and Epidemiology Network (Table 2).

#### Summary findings and reported limitations

This section presents key findings extracted from EWS studies (see Table 3) following the evaluation criteria presented in the methodology and their reported limitations. Most of the studies presented sensitivity, specificity, and NPV/PPV as measurements of the validity of the corresponding models. A range between 40% and 100% for sensitivity and 12.5% to 100% for the PPV have been reported, depending primarily on the type of model and the alarm indicators used. Generally, meteorological indicators were the best performing predictors, but a combination of meteorological and other indicators outperformed the single indicator prediction—i.e., the prediction model improved when other nonclimatic alarm indicators were included to the model containing climate indicators. While several EWSs demonstrated reasonable predictive abilities, 5 models showed outstanding performance using the following alarm indicators: (1) the dynamic risk maps absolute shrinkage and selection operator (LASSO) processing multiple meteorological information (with 1 study adding on epidemiological and entomological alarm indicators) [26]; (2) autoregression integrated moving average (ARIMA) using meteorological information and Google Trends data [40]; (3) Shewhart moving average regression model (SMAR) maintaining a combination of meteorological, epidemiological, and entomological alarm indicators [12,21]; (4) seasonal autoregressive integrated moving average (SARIMA) [35]; and (5) the stochastic Bayesian maximum entropy (BME) model using a mix of meteorological and entomological alarm indicators [37]. A total of 20 EWS studies demonstrated high temporal prediction ability, but only 3 [27,28,38] showed high spatial prediction ability. Several studies reported data- or model-related limitations, with 10 studies indicating issues related to the outbreak information (such as poor or unreliable case reporting, small historical records, bias created by interventions or other confounders, and lack of geotagged weekly records). Three studies [22,41,42] referred to the lack of climate information and biased, inaccessible, or poor resolution data. Other limitations addressed the methodological approach with 3 studies [26,31,35] declaring method-related issues such as that methods (like LASSO) are not amenable to direct interpretation, requiring advanced statistical skills, or have a limited prediction robustness.

Concerning the EWS implementation, the following information has been provided: All studies described the EWS prediction coverage for national and district levels, but only 2 studies [27,38] showed predictions for the subdistrict level. Furthermore, half of the studies (14) featured the possibility of successfully implementing EWS, at national or district levels, but only 5 studies [21,26,28,36,43] showed the feasibility to be integrated into existing national surveillance programs. The users’ perception of EWS was insufficiently assessed in several studies, but 18 indicated the possibility to be used by health managers at the MOH and district levels; 5 studies [32,39,40,44,45] reported the use of EWS being limited to research institutes. The user-friendliness of EWS was reported in only 2 studies [21,31], showing a general satisfaction by users.

### Zika

#### EWS characteristics and features

Studies on Zika were primarily triggered by the 2015 to 2016 Zika virus (ZIKV) epidemic. Thus, only 2 studies were retrieved, which discussed the EWS for Zika outbreaks that matched our selection criteria. Both studies, of which one was an IBS-based and the second was an EBS-based study, applied new algorithms using alarm-informed EWS. Both studies described the need for routinely accessing alarm information for running the tools. One study used both confirmed and suspected cases for early outbreak warning [24], while the other study referred only to suspected cases that reflects the diagnostic and reporting complexity associated with this disease. One study employed social media information (Google Trends search) [24], and another used meteorological information as predictors [25]; however, both studies used the national surveillance programs as sources for processing their data.

#### Summary findings and reported limitations

There was no validity testing presented by both studies, such as sensitivity or specificity. In one study [24], ARIMA was reported as a model to improve the prediction of Zika outbreaks when integrated with Google Trends data. The other study [25] showed the usefulness of humidity, rainfall, and maximum air temperature as alarm indicators for outbreak prediction. As concluded by Teng and colleagues, using dynamic data from Google Trends as indicators can significantly advance the prediction model mainly when more sophisticated statistical models like ARIMA are used [24]. Nevertheless, meteorological alarm indicators have generated high temporal predictions with up to 20 weeks ahead of an outbreak, but both studies failed the spatial prediction. No major limitations were reported, but one study showed a model-related limitation due to the complexity in handling temporal functions in relation to the spatial functions of the model [25].

### Malaria

#### EWS characteristics and features

Out of the 7 EWS malaria studies, 6 used IBS and 1 [46] used an EBS—processing at least 1 meteorological, entomological, or epidemiological indicator. All studies demanded routine access to data, and all but 2 required advanced statistical and analytical skills for practicing or integrating the tool. The outbreak indicators used were hospitalized (1 study) [47] or laboratory-confirmed cases (3 studies) [34,48,49], while 2 studies [46,50] used clinically confirmed cases (hospitalized). Data collection was done on a weekly or monthly basis, and studies demonstrated a spectrum of indicators: All used meteorological and epidemiological information, some entomological (3 studies) [47,48,51], and one study demonstrated the cartographic indicator. None of the studies assessed the user experience with EWS, but one [34] described how the involvement of stakeholders improved the support and institutionalization of the tool; 3 studies [34,46,48] stated that the tool integration into existing epidemiological and climatic data hub created a better environment for further development and use of the tool. All studies used meteorological information as predictors. Surveillance data were obtained from local, regional, or national databases, and meteorological information was obtained from local stations or satellites.

#### Summary findings and reported limitations

Sensitivity and specificity were reported to be high in one study [48], mentioned to be high but not measured in 2 studies [47,49] and varied according to mosquito survival probability and temperature in a fourth study [51]. Several mathematical models were used to create predictions, with the additive and multiplicative models being the ones with high sensitivity and specificity. Several meteorological indicators were used as predictors of which temperature and rainfall were the most frequently used indicators. In general, environmental factors and climate variability correlated with malaria incidence, but no outbreak prediction model has been developed that includes different types of epidemiological, environmental, and meteorological alarm indicators. The alarm indicators have managed to generate a high temporal prediction with up to 6 months ahead of outbreak, particularly if unusual weather conditions like ENSO were concerned. Several limitations were noted: Some models were tested for specific settings (e.g., highlands in Africa), others include only remotely sensed environmental indicators—remote sensing is a useful approach in the context of climate predictions, including flood and earthquake disaster prediction, but tends to lack sufficient evidence for early warning of infectious diseases in general. Thus, when used solely in an early warning, they are viewed as a limitation in the model. Confounding factors that affect malaria risk such as land use/land cover, population mobility, local hydrology, socioeconomic factors, and public health interventions were not captured.

## Discussion

When searching for high-level evidence in the area under investigation, we found 2 systematic reviews [52,53] reporting on predictive modeling tools particularly for dengue. However, we found no high-level evidence on tools applications outside their mathematical modeling or on potential alarm indicators to be used, although these were the prime focus of both reviews. The study by Racloz and colleagues has successfully summarized the benefits of combining various epidemiological tools focusing on the ability to incorporate climatic, environmental, epidemiological, and socioeconomic factors to create an EWS and has outlined optimal prediction models [52]. The second study by Louis and colleagues addressed the risk mapping–related issues and human mobility as promising alarm indicators and maintained a thorough review of their limitations [53]. Besides the structural and statistical features of the tool, our review has additionally addressed essential operational aspects (prediction quality, the implementation, integration, and user perspectives of EWS) and public health implications of EWS, independently for each retrieved disease, keeping in mind that for any outbreak prediction, a reliable surveillance system is essential. For instance, misclassifications, data arriving late, missing values, and human errors during the data entry may compromise the EWS.

### Dengue

All 28 identified dengue studies maintained an IBS approach, and these demanded routine and timely access to surveillance information potentially impacting the effectiveness of early warning tools. Published studies show that consistent and frequent case reporting has stronger predictive capacity, which usually tends to be delayed by monthly reporting schedules [54]. Our results show that weekly reporting of surveillance data correlate with increased sensitivity and PPV; however, only one-third of all dengue studies presented weekly data, negatively impacting the effectiveness and potentially cost-effectiveness of the early warning process [12,21,26–28,31,41,42,44].

A total of 27 dengue studies demonstrated outbreak forecasting, using multiple (9/27) and single alarm (18/27) indicators mostly meteorological ones. However, access to meteorological information on a regular time basis is challenging in several settings [55]. Three studies included regional or international data resources, which, however, demand highly skilled users and advanced digital systems for international data processing [23,33,56].

Furthermore, the mathematical model was identified to affect the predictive ability of EWS due to (1) the choice of the analytical approach employed; and (2) the range of lag times between independent variables and epidemic dengue transmission as demonstrated by Racloz and colleagues and others [52]. Among the retrieved dengue studies, almost all reported estimates on sensitivity, specificity, NPV, or PPV but their prediction performances varied substantially depending on the statistical model used and the data quality. Out of the 19 studies with reported high temporal prediction quality (Table 3), the Bayesian algorithms and generalized linear models were the most prevalent [29,31,36,39,45,57,58]. Additionally, 4 studies used LASSO [22,26,27,45], 3 with ARIMA and time series analysis [28,40,56] independently, and 2 used the Shewhart/endemic channel method [12,21]. Only 4 studies showed evidence of adaptation to less skilled users, which can be significant for public health use [12,21,26,27].

The interaction between changing climate and increasing human mobility as drivers for emerging diseases warrants novel frameworks for assessing the linkage between disease transmission, climate change, and public health intervention in order to reach effective EWS. The use of data mining techniques, such as social media or travel information, in combination with surveillance data has emerged as an alternative source of real-time high-resolution geospatial data on a large scale [59]. However, our review showed minimal evidence of studies exercising such potential alarm indicators, which limits their contribution to outbreak preparedness and response planning [59,60]. The LASSO model is one typical example of such a tool that can potentially contribute to this concept. However, LASSO or similar model concepts demand high-quality big data. As these resources are (a) typically scarce in many countries; and (b) having a tendency to complicate the interpretation of the prediction outputs [26,45], the application in data-constrained settings and by unskilled users is likely to be limited. While limited data accessibility and poor quality have been described by several studies [32,35,41,42,56], published reports highlight the benefits of combining temporal data for analysis of the temporal kinetics and spatial data for the identification of high risk areas [61]. Only 3 retrieved studies [27,28,38] demonstrated high spatial prediction abilities, all attributed to settings with advanced data and surveillance systems. Two of those showed high temporal and spatial prediction, allowing for identification of population at risk at smaller spatial units, which can significantly contribute to targeted vector control [27,38].

### Zika

Since the Zika emergence in 2015, the number of PubMed references for ZIKV has risen from 181 to 516 in 2019, with a high proportion focusing on the consequences of ZIKV infection during pregnancy [62]. Only 2 studies have been identified in this review, with a focus on EWS for Zika outbreaks in the Americas. One study had explored the use of Google Trends data as a predictor [24], and the other used a set of meteorological information for generating outbreak predictions [25]. The use of “suspected cases” as outcome variables in both retrieved studies could possibly be explained by the complexity of the Zika diagnosis and the large number of mild cases [62]. For dengue, the prediction accuracy of EWS was superior when using hospitalized or laboratory-confirmed cases compared to suspected cases [12,21]. However, no measurements of sensitivity or PPV were used in both Zika studies, albeit both ARIMA and generalized linear models predicted up to 20 weeks ahead of the outbreak. Limitations of zika outbreak predictions are not discussed in the respective papers, but are likely to be similar as those shown for dengue predictions.

### Malaria

Since the inception of Roll Back Malaria (RBM) in 1998, it was clear that the early detection, containment, and prevention of malaria epidemics were key elements of the Global Malaria Control Strategy and Malaria Early Warning Systems (MEWS) [63,64]. Some countries have developed epidemic risk monitoring using simple transmission risk indicators such as excess rainfall [65], but only Kenya has published the development and implementation of MEWS [34]. Since then, several studies and initiatives for EWS have been developed; however, the literature to address the tools’ scope of its predictability, implementability, and users’ perspectives are significantly scarce. The 7 studies identified in this review used different mathematical models and different combinations of indicators. Most of the mathematical models found in this review applied time series including additive and multiplicative models [34,46–48], but lacked vulnerability indicators such as low immunity or drug resistance, which might be prevalent in these study settings. Meteorological indicators ranged from common indicators like rainfall and humidity to land surface temperature or vegetation indices obtained through more sophisticated satellite systems that are usually provided by projects or partnerships, of which their sustainability was never assessed. Like the case with dengue, this wide range of indicators is augmented by additional data sources sought from regional or international entities. The development of implementable and user-friendly malaria EWSs, as shown in the reviews by Githeko and colleagues and Merkord and colleagues [34,46], is a key factor for better disease preparedness and timely response activities.

### Public health implications for EWS applications

The EWS tool is primarily aimed at supporting district health managers and national health planners to mitigate or prevent disease outbreaks, ideally using tools that are integrated in the national surveillance programs [66,67]. To further ensure effective functions, EWS should conceptually be perceived as an information system designed to support the decision-making of national- and local-level institutions but also enable vulnerable groups in the society to take actions to mitigate the impacts of an impending risk. As apparent from this review, users of current EWSs were mostly from the central (MOH) levels, with only few tools facilitating district-level applications. The integration of EWS into existing national surveillance program was marginal with only 5 studies [12,21,26,27,34], out of 37, demonstrating their experience of integration (Table 1). Furthermore, the majority of studies have not assessed the feasibility of implementing EWS into national programs, and a few studies have declared the need for high-skilled users and resources—2 limiting factors that are unlikely to exist at small spatial levels in settings where disease outbreaks are public health burdens. Nevertheless, there is an observed trend toward applications of more advanced statistical models with higher predictive abilities that can further advance the prediction and control of disease outbreaks. Generally, early warning and response system that are capable of demonstrating evidence of prospective predictive ability and allows technical and practical adaptations of local public health responses while augmenting communications channels between users at central and district levels are tools that are more likely to be implemented into national surveillance programs. Advancing into frameworks that can facilitate at a low-cost IT maintenance and adapted to unskilled users are features of tools that are plausibly integrable into existing national systems.

As shown in the method section, the IBS and EBS are 2 main channels of information for a functioning EWS. Almost all studies reviewed in this paper maintained an IBS type of application to support EWS, with the exception of 1 Zika- and 1 malaria-related study using the EBS approach [24,46]. The combined use of both could potentially include other sources of information, such as sources from outside the health sector, which is the prime concept of the EBS. With the majority being of IBS-based EWS, the applications of the forecasting tools tend to be less efficient and deviate from the epidemic intelligence concept—the systematic collection, analysis, and communication of any information to detect, verify, assess, and investigate events and health risks with an early warning objective, as opposed to monitoring of disease trends or burdens [68]—which ideally combines both IBS and EBS for more robust outbreak detection.

Meteorological indicators are key predictors, but they are often inaccessible on a timely basis for health services managing the EWS. Benefiting from multiple assessments of users’ perspectives while defining the tool end users, countries like Mexico and Brazil, for instance, have managed to recognize the essence of the availability of local meteorological stations and have therefore organized an improved access to meteorological information [21,29,36,58,69].

Our literature review has identified very limited information for chikungunya and yellow fever outbreaks prediction, none of them fulfilling the inclusion criteria. Studies of EWS for yellow fever outbreaks are limited probably due to the existence of vaccines for this disease. However, chikungunya has now expanded from Africa, Latin America, and Asia to the European region [70,71]. It is quite worrying that studies on EWS tools for diseases like chikungunya are scarce. Although many EWS rely on meteorological information, only 3 studies [28,29,47] performed a prospective type analysis of early warning performance, with no rigorous study could be found.

### Limitations of our study

The authors decided to include EWS on malaria to broaden the scope and eventually learn mutually from applications of different fields of disease control (*Aedes* borne versus *Anopheles* borne), but also by including yellow fever (vaccine preventable) and other vector-borne disease (mainly relying on vector control interventions). By virtue of their variability in terminology and definitions as well as difficulties in synthesis, the format of a scoping review was applied as a form of review design. Nevertheless, this scoping review follows the PRISMA criteria for conducting a systematic review without performing rigorous critical appraisal of included studies due to time constraints. However, we think that by including only peer-reviewed papers focusing on implemented EWSs or those under development provides a certain guarantee for high-quality papers, which will be an added value to the existing literature. The search strategy was limited to the target diseases not including search terms as “arboviruses” or “febrile illnesses” or “priority diseases” or others, which might be potentially relevant to the review, considering the broadness of the scoping review, Furthermore, stakeholders have been occasionally involved during the 14-month duration of this scoping process—during an Andean regional meeting in Bogota and in Colombia with an expert panel in charge of EWSs. Another limitation might be that we may have missed a few relevant studies; however, by including the Google Scholar search engine, we could overcome this issue, assuming that relevant papers could be identified, which were not indexed in other databases like PubMed. Due to the language barrier, we were unable to include publications in Chinese language, but several English papers from China were included, which could adequately represent the rich experience from the Chinese context.

### Conclusions

This scoping review demonstrated gaps and challenges related to the structural, statistical, and operational designs of EWS, and these varied per disease and their corresponding settings. The country surveillance system is an integral part in the overall early warning process where the lack of accessibility to timely and quality data is crucial for establishing a reasonable EWS. Nevertheless, a substantial number of studies (except for dengue) failed to demonstrate any predictive power mainly that predictions based on complicated statistical models are difficult to carry out in low- and middle-income countries. This review has furthermore revealed a significant gap in effectively evaluating the role of EWS in the disease outbreak prediction and control given that the majority of EWS assessment studies have primarily been of retrospective designs. The lack of tool assessments regarding the implementation into existing routine surveillance as well as the feasibility of translating model outputs into local vector control and action plans will unlikely support the global health agenda for controlling disease outbreaks. Likewise, the missing user perspectives in the retrieved studies signals shows that most of the EWSs remain in the academic environment, and little effort has been spent on testing their effectiveness or cost-effectiveness in reducing disease outbreaks. Collectively, findings from this review claim the need for more pragmatic and context-adapted EWS tools, which address the user perspectives and its effectiveness in predicting outbreaks in local settings and trigger response activities.

**Key learning points**

- Only minimal studies have addressed the early warning system (EWS) users’ perspectives with significant lack of implementation research assessments of EWS for chikungunya, dengue, malaria, yellow fever, and Zika outbreaks.
- While the majority of studies have focused on the development and applications of the temporal prediction of the EWS, the spatial analysis of the disease prediction is crucial for effective vector control and response but rarely discussed or assessed in the literature.
- The EWSs should be viewed as frameworks for improving the coordination of the overall disease outbreak control and response where full stakeholder involvement and assessment are warranted.

**Top five papers**

1. Chen Y, Chu CW, Chen MIC, Cook AR. The utility of LASSO-based models for real time forecasts of endemic infectious diseases: A cross country comparison. J Biomed Inform. 2018;81:16–30. doi: 10.1016/j.jbi.2018.02.014
2. Liu-Helmersson J, Quam M, Wilder-Smith A, Stenlund H, Ebi K, Massad E, et al. Climate Change and Aedes Vectors: 21st Century Projections for Dengue Transmission in Europe. EBioMedicine. 2016;7:267–77. doi: 10.1016/j.ebiom.2016.03.046
3. Semenza JC. Prototype Early Warning Systems for Vector-Borne Diseases in Europe. Int J Environ Res Public Health. 2015;12:6333–51. doi: 10.3390/ijerph120606333
4. Racloz V, Ramsey R, Tong S, Hu W. Surveillance of Dengue Fever Virus: A Review of Epidemiological Models and Early Warning Systems. PLoS Negl Trop Dis. 2012;6:e1648. doi: 10.1371/journal.pntd.0001648
5. Louis VR, Phalkey R, Horstick O, Ratanawong P, Wilder-Smith A, Tozan Y, et al. Modeling tools for dengue risk mapping—a systematic review. Int J Health Geogr. 2014;13:50. doi: 10.1186/1476-072X-13-50

## Supporting information

S1 Text Country distribution of included studies. (DOCX)
