# Supplementary material for: Early warning systems (EWSs) for chikungunya, dengue, malaria, yellow fever, and Zika outbreaks: What is the evidence? A scoping review
Source: PLoS Negl Trop Dis. 2021 Sep 16;15(9):e0009686. doi: 10.1371/journal.pntd.0009686 (PMC8445439; doi:10.1371/journal.pntd.0009686)
Supplement: S1 Text — (DOCX) [file pntd.0009686.s001.docx]

**China**

**D5** [25]**:** 2014. The coverage of the tool is at district- or province-level. Tool is part of existing China Infectious Disease Automated-alert and Response System (CIDARS). Initial phase of real-world implementation nationwide. The end-user is county- and MOH-level.

**D11** [39]**:** 2014. The coverage of the tool is at district- or province-level. Tool is aimed at the national disease surveillance system and meteorological agency. Tool implementation Pilot plan at provincial-district- and regional-levels. The end-user is China CDC and provincial and districts users.

**D12** [37]**:** 2016. The coverage of the tool is at district- or province-level. The tool integration into national surveillance system is not explored. Tool implementation at district-level. The end-user is local district managers.

**D13** [38]**:** 2017. The coverage of the tool is at district- or province-level. The tool integration into national surveillance system is not explored. Tool implementation is possible nut requires substantial resources and skilled staffs. The end-user is county-level.

**D16** [41]**:** 2017. The coverage of the tool is at provincial- and district-level. The tool integration into national surveillance system is not explored. The tool implementation is at district- and provincial-levels. The end users are researchers at institute level.

**Brazil ^a, b^**

**D22** [58]**:** 2011, The coverage of the tool is at district-level. Integration into national surveillance is not assessed. The tool can be implemented at national (MOH) level. End-users are public health managers.

**D24** [32]**:** 2016, The coverage of the tool is at district-level. Integration into national surveillance is not assessed. The tool can be implemented at national (MOH) level. End-users are public health managers.

**D25** [67]**:** 2014, The coverage of the tool is at district-level. Integration into national surveillance is not assessed. The tool can be implemented at national (MOH) level. End-users are public health managers.

**D26** [30]**:** 2013, The coverage of the tool is at district-level. Integration into the country climate and health observatory system. The tool can be implemented at municipality level. End-users are public health managers.

**PAHO Region**

**Z1** [42]: 2017. The coverage of the tool is at regional-level. The tool integration into national surveillance system is not explored. The tool implementation is at not assessed. The end users are public health managers.

**Cambodia**

**D4** [24]**:** 2019. The coverage of the tool is at district- or province-level. Leve of Integration was not explored. Cost-free and easy to implement at the provincial level. The end-user is provincial- and national-level.

**Dominican Rep**

**D3 ^b^** [12]: 2016. The coverage of the tool is at district- or province-level. Integration into national surveillance is possible but not discussed in details. The tool can be implemented at district-, national- and regional-levels. The end-user is district managers, regional- and central-level.

**Colombia ^e^**

**D9** [33]: 2017. The coverage of the tool is at sub-district-level. The tool integration into national surveillance system is not explored. The tool implementation is not explored. The end users of the tool are not explored.

**D15** [35]: 2014. The coverage of the tool is at provincial- and district-level. The tool integration into national surveillance system is not explored. The tool implementation is not explored. The end users are researchers at institute level.

**Z2** [43]: 2018. The coverage of the tool is at district-level. The tool integration into national surveillance system is not explored. The tool implementation is not assessed. The end users are not assessed.

**M6** [40]: 2006. The coverage of the tool is at province-level. The tool integration into national surveillance system is not explored. The tool implementation is not assessed. The end users are not assessed.

**Indonesia**

**D7** [57]: 2016. The coverage of the tool is at district-level. The tool integration into national surveillance system is not explored. The tool implementation is not explored. The end users of the tool are not explored.

**D19** [27]: 2017. The coverage of the tool is at district-level. The tool integration into national surveillance system is not explored. The tool implementation is not explored. The end users is researchers at institute level.

**D20** [55]: 2012. The coverage of the tool is at district-level. The tool integration into national surveillance system is not explored. The tool implementation is at national-level. The end users are public health officers.

**D21** [34]: 2008. The coverage of the tool is at district-level. The tool integration into national surveillance system is not explored. The tool implementation is not assessed. The tool proposed public health managers as end users.

**S1 Text. Country distribution of included studies**

**Europe**

**D10** [23]: 2015. The coverage of the tool is at national-level. The tool integration into national surveillance system is not explored. The tool implementation is not explored. The end users of the tool are at regional level.

**Uganda ^d^** [46]

**Vietnam ^b^** [12]

**Malaysia ^a, b^** [12, 21]

**Ethiopia**

**M4** [48]**:** 2012. The coverage of the tool is at province level. The tool integration into national surveillance system is not explored. The tool implementation is at district-levels. The end users are not assessed

**M7** [44]**:** 2017. The coverage of the tool is at province level. The tool integration into national surveillance system is not explored. The tool implementation is at provincial-level. The end users are public health officials

**Tanzania ^d^** [46]

**Japan**

**D28 ^c^** [22]: 2018. The coverage of the tool is at district-level. The tool integration into national surveillance system is not explored. The tool implementation is at district level. The end users are public health managers.

**Kenia**

**M1** [45]**:** 2001. The coverage of the tool is at district-level. The tool integration into national surveillance system is not explored. The tool implementation is at district-level. The end users are health personnel and decision makers.

**M2 ^d^** [46]**:** 2014. The coverage of the tool is at district-level. The tool integration into national surveillance system is not explored. The tool implementation is at district-level. The end users are public health officers.

**M3** [47]**:** 2018. The coverage of the tool is at district-level. The tool integration into national surveillance system is not explored. The tool implementation is at district-level. The end users are malaria control and health district officers.

**Singapore ^c^**

**D2** [28]**:** 2016. The coverage of the tool is at district- or province-level. The tool is an integral part of Singapore’s dengue control program-tool. The tool can be implemented at national (MOH) level. The end users if the MOH.

**D6** [36]: 2018. The coverage of the tool is at sub-district-level. The tool integration into national surveillance system is not explored. The tool implementation is not explored. The end users is not explored.

**D17** [57]**:** 2012. The coverage of the tool is at national-level. The tool integration into national surveillance system is not explored. The tool implementation is at district-levels. The end users are district health managers.

**Cuba**

**D8** [29]: 2015. The coverage of the tool is at national-level. The tool integration into national surveillance system is not explored. The tool implementation is not explored. The end users of the tool are not explored.

**French Guiana**

**D14** [40]: 2016. The coverage of the tool is at national-level. The tool integration into national surveillance system is not explored. The tool implementation is not explored. The end users are researchers at institute level.

**Sri Lanka**

**D27** [56]: 2018. The coverage of the tool is at district-level. The tool integration is not explored and its implementation is at district level. The end users are public health managers.

**Taiwan ^c^**

**D23** [31]: 2011. The coverage of the tool is at district-level. The tool integration into national surveillance system is not explored. The tool implementation is at MOH level. The end users are not assessed.

**Thailand ^c^**

**D18** [26]**:** 2012, The coverage of the tool is at district-level. The tool integration into national surveillance system is not explored. The tool implementation is not explored. The end users are researchers at institute level.

**Mexico ^b^**

**D1^a^** [21]: 2018, The coverage of the tool is at district- or province-level. Integration into national surveillance was evident from Mexico! The tool can be implemented at national, regional and local-area. End-users are district managers, regional-level and central-level.

**Solomon Islands**

**M5** [49]**:** 2017. The coverage of the tool is at province level. The tool integration into national surveillance system is not explored. The tool implementation is at provincial and district-levels. The end users are meteorological and health services officials.

a: countries part of study D1 (Brazil, Mexico, Malaysia)

b: countries part of study D3 (Brazil, Dominican Republic, Mexico, Malaysia, Vietnam)

c: countries part of study D28 (Japan, Thailand, Singapore, Taiwan)

d: countries part of study M2 (Kenya, Tanzania, Uganda)
